# Report of Mortality in Buffalo for the Month March, 1856

**Published:** 1856-05

**Authors:** C. L. Dayton

**Affiliations:** Health Physician


ART. VII.—*Report of Mortality in Buffalo for the month March*, 1856.
By C. L. DAYTON, M. D., Health Physician.

| DISEASES. | No. | Males. | Females. |
|---|---|---|---|
| Apoplexia, | 1 | .... | 1 |
| Accidens, | 5 | 4 | 1 |
| Bright's Disease, | 1 | 1 | .... |
| Bronchitis, | 1 | 1 | .... |
| Cancer, | 1 | .... | 1 |
| " of the Womb, | 2 | .... | 2 |
| Concussio Cerebri, | 1 | .... | 1 |
| Contusio " | 1 | .... | 1 |
| Cynanche Tonsillaris, | 1 | 1 | .... |
| " Trachealis, | 1 | 1 | .... |
| Diarrhœa Chronica, | 1 | .... | 1 |
| Dentitio, | 1 | .... | 1 |
| Erysipelas, | 2 | 2 | .... |
| Eclampsia, | 17 | 8 | 8 |
| Febris Typhoid, | 2 | 2 | .... |
| " Scarlatina, | 5 | 4 | 1 |
| " Nervosa, | 1 | 1 | .... |
| Hydrocephalus, | 6 | 4 | 2 |
| Hydrops, | 1 | .... | 1 |
| Hyperæmia Cerebralis, | 1 | 1 | .... |
| Meningitis, | 1 | .... | 1 |
| Neuralgia, | 1 | .... | 1 |
| Poisoned, | 1 | .... | 1 |
| Pleuro Pneumonia, | 2 | .... | 2 |
| Paralysis Pulmonalis, | 1 | 1 | .... |
| Pneumonitis, | 7 | 4 | 3 |
| " Typhoides, | 2 | 2 | ... |
| Pertussis, | 1 | 1 | .... |
| Rheumatic Carditis, | 1 | 1 | .... |
| Senectus, | 4 | 4 | .... |
| Still-born, | 7 | 4 | 1 |
| Tabes Mesenterica, | 1 | .... | 1 |
| Tuberculosis Pulmonalis, | 17 | 4 | 12 |
| Ignotus, | 13 | 8 | 5 |
| Totals, | 111 | | |

SEXES.

| | |
|---|---|
| Males, | 59 |
| Females, | 48 |
| Sex not given, | 4 |
| Total, | 111 |

REGISTER OF MORTALITY—Continued.

AGES.

| | | | |
|---|---|---|---|
| Still-born, | 7 | Between 10 years and 20 years, | 3 |
| 1 day, | 5 | " 20 " " 30 " | 9 |
| Between 1 day and 30 days, | 6 | " 30 " " 40 " | 12 |
| " 1 month and 6 months, | 10 | " 40 " " 50 " | 12 |
| " 6 months and 12 " | 4 | " 50 " " 60 " | 3 |
| " 1 year " 3 years, | 19 | " 60 " " 70 " | 3 |
| " 3 " " 5 " | 5 | " 70 " " 80 " | 3 |
| " 5 " " 10 " | 4 | " 80 " " 90 " | 0 |
| Total number under 10 years, | 60 | Total number over 10 years, | 45 |
| | | Ages not given, 6. Total, | 111 |

NATIVITIES.

| | | | |
|---|---|---|---|
| American, (white, 60) colored, 1, | 61 | French, | 4 |
| German, | 18 | Swiss, | 0 |
| Irish, | 16 | Norwegian, | 0 |
| English, | 1 | Country not given, | 9 |
| Canadian, | 2 | | |
| Total, | | | 111 |

